# Zhi-Zi-Chi Decoction Alleviates Depressive-like Behaviors by Regulating Gut Microbiota and Targeting the AMPK/PI3K-TOR Pathway via Its Metabolite Protocatechuic Acid

**DOI:** 10.3390/ph19060819

**Published:** 2026-05-23

**Authors:** Xue Jiang, Jicheng Yang, Ying Zhang, Yusheng Zhang, Qingqing Li, Shaoqi Song, Zhen Ouyang, Hongjun Yang, Xianyu Li, Luqi Huang

**Affiliations:** 1 School of Food and Biological Engineering, Jiangsu University, Zhenjiang 212013, China; jiangxuexue941213@outlook.com (X.J.); zhenouyang@ujs.edu.cn (Z.O.); 2 Beijing Key Laboratory of China Academy of Chinese Medical Sciences on Prevention and Treatment for Major Diseases, Experimental Research Center, China Academy of Chinese Medical Sciences, Beijing 100700, China; yjc18143609206@163.com (J.Y.); zhangying200236@163.com (Y.Z.); yushengzhang271727@foxmail.com (Y.Z.); lqq5213@126.com (Q.L.); m18053676639@163.com (S.S.); 3 State Key Laboratory for Quality Ensurance and Sustainable Use of Dao-di Herbs, China Academy of Chinese Medical Sciences, Beijing 100029, China; hongjun0420@vip.sina.com; 4 Institute of Chinese Materia Medica, China Academy of Chinese Medical Sciences, Beijing 100700, China

**Keywords:** anti-depression, gut–brain axis, protocatechuic acid, chronic restraint stress, gut microbiota, Zhi-Zi-Chi decoction

## Abstract

**Background:** Neuroinflammation and gut–brain axis (GBX) dysregulation are key pathological drivers of stress-related neuropsychiatric disorders. Zhi-Zi-Chi Decoction (ZZCD), a classic Traditional Chinese Medicine (TCM) formula, has been clinically used to alleviate mental disturbances via the TCM principle of “clearing heat and relieving restlessness.” Still, its modern neuroprotective mechanisms, especially its links to gut microbiota and central signaling pathways, remain incompletely elucidated. **Purpose:** This study aimed to systematically investigate the therapeutic effects of ZZCD on chronic restraint stress (CRS)-induced neurodysfunction in mice and clarify its mechanisms from the perspectives of TCM theory, material basis, gut microbiota–metabolite axis, and central signaling pathways. **Method:** CRS mice were treated with ZZCD or protocatechuic acid. Behavioral tests evaluated depression- and anxiety-like behaviors. UHPLC-Q-TOF/MS identified ZZCD’s chemical constituents; 16S rRNA sequencing and untargeted metabolomics analyzed gut microbiota and metabolite changes. Western blot, immunofluorescence, and proteomics examined neuroinflammation, microglial polarization, and signaling pathway activity (PI3K/Akt/mTOR, AMPK). **Results:** ZZCD reversed CRS-induced depression- and anxiety-like behaviors and suppressed neuroinflammation. Mechanistically, UHPLC-Q-TOF/MS identified 424 ZZCD constituents, with prenol lipids, organooxygen compounds, and flavonoids as the most abundant. ZZCD reversed CRS-induced imbalance in gut microbiota, reducing pro-inflammatory Prevotella and enriching beneficial Lactobacillus, and mediated the enrichment of the prebiotic metabolite PCA in colonic and serum samples, which crossed the blood–brain barrier (BBB) to exert neuroprotection. Additionally, ZZCD and PCA normalized the PI3K/Akt/mTOR pathway and activated AMPK, promoting M2 microglial polarization and restoring synaptic plasticity. **Conclusions:** ZZCD exerts antidepressant effects by a gut-microbiota-dependent modulation of PCA-PI3K/Akt/mTOR and AMPK dual axes that converts microglia from M1 to M2, providing ethnopharmacological evidence and a mechanistic rationale for its clinical application in major depressive disorder.

## 1. Introduction

Major Depressive Disorder (MDD) is clinically characterized by three core hallmarks: cognitive-somatic dysfunction, anhedonia (loss of interest), and a persistently depressed mood. Epidemiologically, the global lifetime prevalence of MDD is approximately 12%, and it exhibits a remarkably high recurrence rate, with 50% of patients experiencing a relapse within five years of their first episode [1]. Its current first-line therapies mainly contain fluoxetine, sertraline, and escitalopram, while these exhibit limitations, including delayed onset (2–4 weeks), high recurrence rates (~50% within five years), treatment resistance (~30% of patients), and adverse effects (GI distress, sexual dysfunction) [2]. Therefore, the discovery and development of novel therapeutic agents and strategies for depression are of paramount importance.

Contemporary research has shifted the understanding of depression beyond a purely emotional disorder; instead, it is now recognized as a systemic “inflammatory brain disease”, in which microglia play a central regulatory role [3]. Consistent evidence from clinical studies demonstrates elevated levels of pro-inflammatory cytokines, specifically interleukin-1beta (IL-1β), interleukin-6 (IL-6), and tumor necrosis factor alpha (TNF-α), in both the peripheral circulation and the central nervous system (CNS) of MDD patients. These pro-inflammatory cytokines can disrupt the integrity of the blood–brain barrier (BBB), a key event that triggers microglial polarization toward the M1 pro-inflammatory phenotype [4]. Overactivation of M1-polarized microglia subsequently induces the release of cytotoxic mediators, including reactive oxygen species (ROS), glutamate, and nitric oxide (NO). These factors collectively contribute to three critical pathological processes: (1) neuronal excitotoxicity, (2) impairment of hippocampal neurogenesis, and (3) attenuation of brain-derived neurotrophic factor (BDNF) signaling. Notably, all three processes have been robustly linked to the severity and chronicity of depressive symptoms in MDD [5,6,7]. Conversely, preclinical studies have provided promising evidence for targeted anti-inflammatory interventions. For instance, redirecting microglial polarization from the M1 pro-inflammatory state toward the M2 anti-inflammatory phenotype, or pharmacologically targeting the IL-1/IL-6/NLRP3 inflammatory pathway, has been shown to rapidly reverse depressive-like behaviors in animal models of depression [7].

Therefore, regulating microglial activity to mitigate neuroinflammation represents a promising therapeutic strategy for depression. Recent preclinical investigations and preliminary clinical studies have demonstrated that several traditional Chinese medicines (TCMs), including Ganoderma lucidum polysaccharides [8], Bupleurum saponins [9], Huanglian Wendan decoction [10], and ginsenoside Rd [11], exert multiple beneficial effects. They effectively inhibit microglial M1 polarization, suppress the activity of the NLR Family Pyrin Domain Containing 3 (NLRP3) inflammasome, reduce hippocampal levels of tumor necrosis factor alpha (TNF-α), interleukin-1beta (IL-1β), and interleukin-18 (IL-18), and restore brain-derived neurotrophic factor (BDNF)-mediated synaptic plasticity. Significantly, these plant-derived extracts can rapidly induce and sustain antidepressant or anxiolytic effects that are comparable to, or even stronger than, those of fluoxetine with no significant adverse effects. This evidence highlights the translational potential of these novel microglia-modulating therapies for mood disorders.

A burgeoning target for depression interventions is the microbiota–gut–brain (MGB) axis, a conceptual framework proposed to explain bidirectional interactions between the gut microbiota and the central nervous system [12]. Clinical and preclinical studies have shown that reduced gut microbial diversity and a decline in short-chain fatty acid (SCFA)-producing bacteria impair intestinal barrier integrity. This barrier disruption permits the translocation of bacterial lipopolysaccharides (LPS) and peripheral pro-inflammatory cytokines into the brain [13,14]. Once in the central nervous system, these peripheral inflammatory inputs drive microglial M1 polarization via the Toll-like receptor 4 (TLR4)/nuclear factor kappa B (NF-κB) and NLRP3 inflammasome pathways. This polarization, in turn, leads to excessive synaptic pruning and decreased hippocampal BDNF levels, the key pathological features linked to depressive phenotypes [15]. In support of the MGB axis’s role in depression, experimental models have shown that germ-free (GF) mice or mice treated with antibiotics exhibit immature, less reactive microglia and display resistance to stress-induced despair-like behaviors. Conversely, fecal microbiota transplantation (FMT) from donors subjected to chronic stress rapidly induces microglial activation and depression-like behaviors in recipient mice. In contrast, interventions such as probiotic supplementation or high-fiber diets, both of which enhance gut SCFA production, have been shown to restore SCFA levels and inhibit microglial activation [16]. Collectively, these findings indicate that microglia act as core effectors of gut-derived inflammatory signals in the pathogenesis of depression, positioning the microbiota–microglia axis as a priority target for the development of novel dietary, probiotic, or pharmacological antidepressant strategies.

Zhi-Zi-Chi decoction (ZZCD) is a classic traditional Chinese medicine (TCM) formulation that combines *Gardenia jasminoides* J. Ellis (recorded in the Chinese Pharmacopeia as Gardenia Fructus) and *Glycine max* (L.). Merr (recorded in the Chinese Pharmacopeia as Semen Sojae Praeparatum). It is a classical formula from *Shanghan Lun* for “clearing heat and relieving restlessness,” clinically used for irritability, insomnia, and depression. Modern phytochemical studies have identified its bioactive components (geniposide from Gardeniae Fructus and isoflavones from Semen Sojae Praeparatum) as neuroprotective and anti-inflammatory agents that modulate microglial activity [17,18]. This formulation was first recorded in the Shanghan Lun, which was usually used to treat restlessness and insomnia [19]. In modern pharmacy, it has been repositioned as a potential antidepressant targeting microglia [20]. Modern phytochemical research has identified geniposide in Gardeniae Fructus [21] and isoflavones in Semen Sojae Praeparatum as the primary bioactive components for neuroprotection, anti-inflammation, antioxidation, antidepressant-like effects, and immune regulation [22,23]. At clinically relevant doses, these components exert multiple neuroprotective effects by inhibiting microglial M1 polarization, suppressing NLRP3 (NLR family pyrin domain-containing 3) inflammasome activation [24], and restoring BDNF/TrkB signaling in the hippocampus [25]. Beyond central nervous system effects, ZZCD simultaneously normalizes aberrant BDNF, TNF-α, pro-inflammatory cytokines and neurotransmitters throughout the gut–peripheral–brain axis while reshaping the gut microbiota—boosting SCFA-producing and anti-inflammatory taxa, curbing pro-inflammatory and tryptophan-metabolizing bacteria, and restoring cecal butyrate depleted by chronic unpredictable mild stress [26]. However, ZZCD regulates gut microbiota and their metabolites to mediate antidepressant effects by modulating microglial phenotype, and the underlying mechanism remains unclear.

In this study, we aims (i) to evaluate the effect of varying doses of ZZCD (low dose: 2 g·kg^−1^·day^−1^; medium dose: 4 g·kg^−1^·day^−1^; high dose: 6 g·kg^−1^·day^−1^) on CRS (chronic restraint stress) mice to verify whether ZZCD can alleviate CRS-induced depression- and anxiety-like behaviors; (ii) to identify how ZZCD regulate gut microbiota, intestine and serum metabolites, and brain protein expression, integrating metagenomic, metabolome, and proteomic; (iii) to validate which gut microbiota can transform the ZZCD to prebiotic metabolites (PCA) involving anti-depression and anti-anxiety behavior; and (iv) to illustrate the pharmacological mechanism of PCA on CRS mice.

## 2. Result

### 2.1. Chemical Profiles of ZZCD

**Figure 1:**
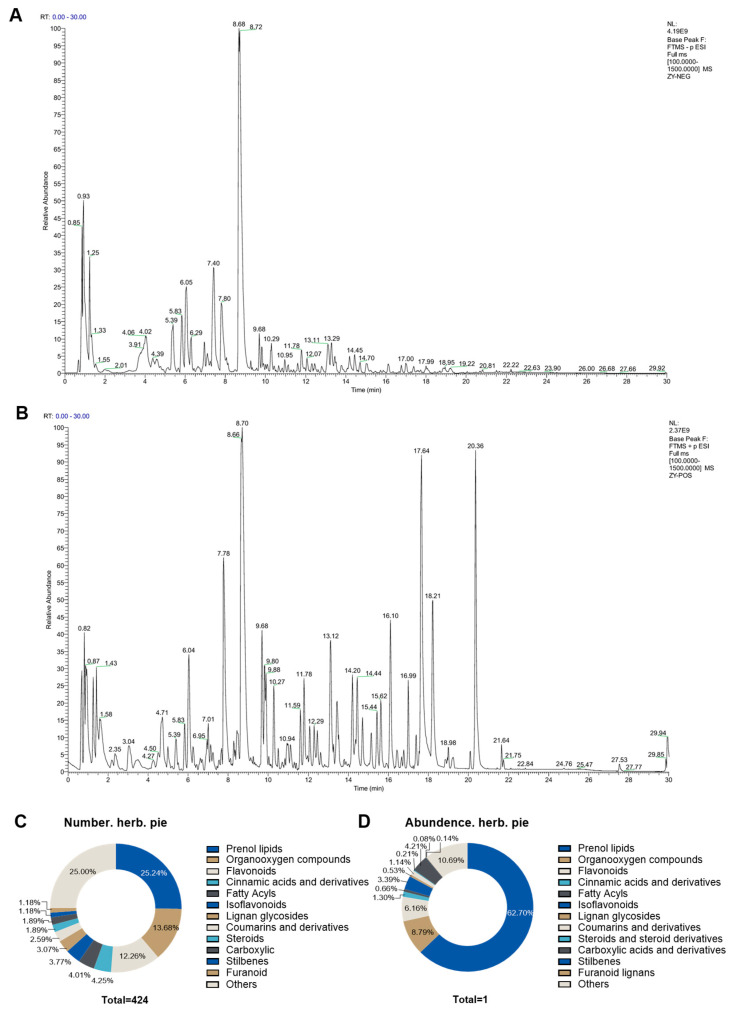
Analysis of ZZCD components using UPLC-QTF-MS/MS. (**A**) TIC of ZZCD in positive-ion scan. (**B**) TIC of ZZCD in negative-ion scan. (**C**) The number of different types of compounds. (**D**) The relative peak area of various types of compounds.

Chemical characterization of ZZCD extract was achieved using UPLC-Q-TOF-MS. The components were tentatively identified based on their MS and MS/MS fragmentation patterns by searching relevant databases and references. The representative total ion chromatograms of the extract detected in both positive and negative ion modes are shown in Figure 1A,B and Supplementary Table S1. In total, 424 chemical components were characterized: 103 in negative ion mode, 190 in positive ion mode, and 128 in both negative and positive ion modes. Details of the identified compounds mainly include 107 of prenol lipids (25.24%), 58 of organooxygen compounds (13.68%), 52 of flavonoids (12.26%), 18 of cinnamic acids and derivatives (4.25%), 17 of fatty acyls (4.01%), 16 of isoflavonoids (3.77%), 13 of lignan glycosides (3.07%), 11 of coumarins and derivatives (2.59%), 8 of steroids (1.89%), 8 of carboxylic (1.89%), 5 of stilbenes (1.18%), 5 of furanoid (1.18%) and 104 of other compound (25.00%), in which relative peak area are 62.70%, 8.80%, 6.16%, 1.30%, 0.66%, 0.53%, 1.14%, 0.21%, 1.21%, 0.08%, 0.14% and 10.69%, respectively (Figure 1C,D). Among them, prenol lipids have a relative abundance of more than 62.7% (Table S2). Previous research showed that isoflavones (e.g., daidzein and genistein from *Semen Sojae Praeparatum*) and geniposide (an iridoid glycoside from *Gardeniae Fructus*) are proposed as the principal bioactive components with neuroprotective and anti-inflammatory activities [25,27]. These results revealed that prenol lipids, organo-oxygen compounds, and flavonoids may be potential components for neuroprotective and anti-inflammatory activities.

### 2.2. Effects of ZZCD on the Anxiety and Depression-like Behaviors of Mice

**Figure 2:**
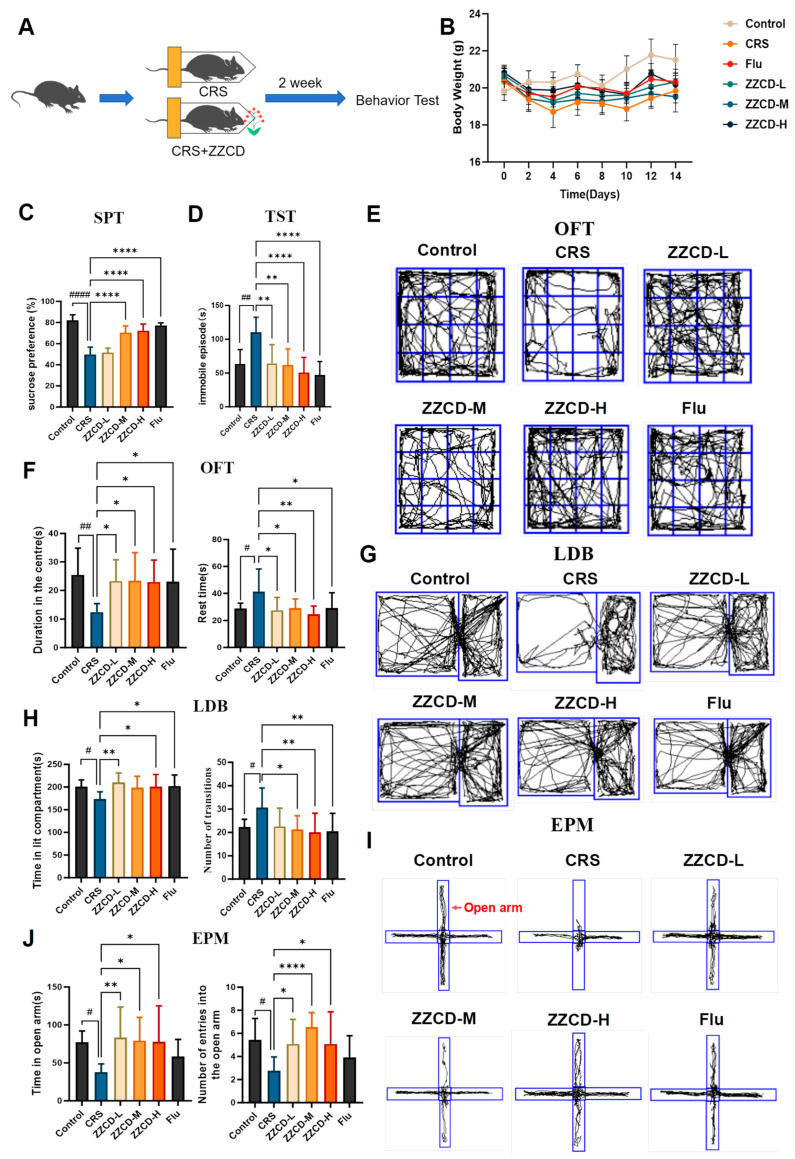
ZZCD prevents CRS-induced behavioral changes. (**A**) Schematic of the experimental paradigm. (**B**) Changes in mouse body weight during CRS. (**C**) Mean sucrose preference (%) in the sucrose preference test (SPT). (**D**) Immobility time in the tail suspension test (TST) in mice. (**E**) Trajectories of mouse movement in the Con, CRS, low-dose (ZZCD-L), middle-dose (ZZCD-M), high-dose ZZCD (ZZCD-H), and fluoxetine (Flx) groups in OFT. (**F**) Rest time and the time spent in the central zone of the open field test (OFT). (**G**) Comparison of movement trajectory in LDB. (**H**) The amount of time mice spent in the light box and the number of times they switched between the light and dark boxes. (**I**) Comparison of movement trajectory in EPM with the vertical grid on the open arm. (**J**) The amount of time mice spent in the open arm and the number of times they entered the open arm. Data are expressed as mean ± SEM. *n* = 12. ^#^ *p* < 0.05, ^##^ *p* < 0.01, ^####^ *p* < 0.0001, compared to the Control group; * *p* < 0.05, ** *p* < 0.01, **** *p* < 0.0001, compared to the CRS group.

A chronic restraint stress (CRS)-induced mouse model was used to evaluate the anxiolytic and antidepressant activities of Zhi-Zi-Chi Decoction (ZZCD). From the initiation of CRS modeling to the completion of behavioral testing, mice subjected to CRS were administered daily oral treatments as follows: ZZCD at three different doses (2 g·kg^−1^·day^−1^, 4 g·kg^−1^·day^−1^, or 6 g·kg^−1^·day^−1^), the positive control drug fluoxetine (Flx), or a vehicle (Figure 2A). Consistent with previous reports, CRS induced weight loss and hedonic deficiency in mice, as demonstrated by a significant reduction in average sucrose preference (Figure 2B,C). Additionally, CRS increased immobility time in the tail suspension test (TST; Figure 2D) and impaired motor activity in the open field test (OFT), evidenced by increased resting time and decreased dwell time in the central region (Figure 2E,F). CRS also promoted the development of anxiety-related behaviors. In the light–dark box (LDB) test, CRS-exposed mice spent less time in the light box and made more transitions between the light and dark compartments (Figure 2G,H). In the elevated plus maze (EPM) test, CRS shortened both the time spent in the open arms and the number of entries into the open arms (Figure 2I,J). As hypothesized, ZZCD treatment reversed CRS-induced reduction in sucrose preference in mice and partially preserved body weight during the modeling period (Figure 2B,C). Compared with vehicle-treated CRS mice, mice in the mid-dose and high-dose ZZCD groups showed reduced immobility in the TST and spent more time in the central zone of the OFT with shorter resting periods (Figure 2D–F). Furthermore, in anxiety-related behavioral assessments (LDB and EPM tests), ZZCD treatment significantly ameliorated anxiety-like behaviors in CRS-exposed mice (Figure 2G–J). Collectively, these findings confirm the anxiolytic and antidepressant properties of ZZCD.

### 2.3. ZZCD Reduced Neuroinflammation and Increased the Number of M2-Type Microglia in the Brains of CRS Mice

**Figure 3:**
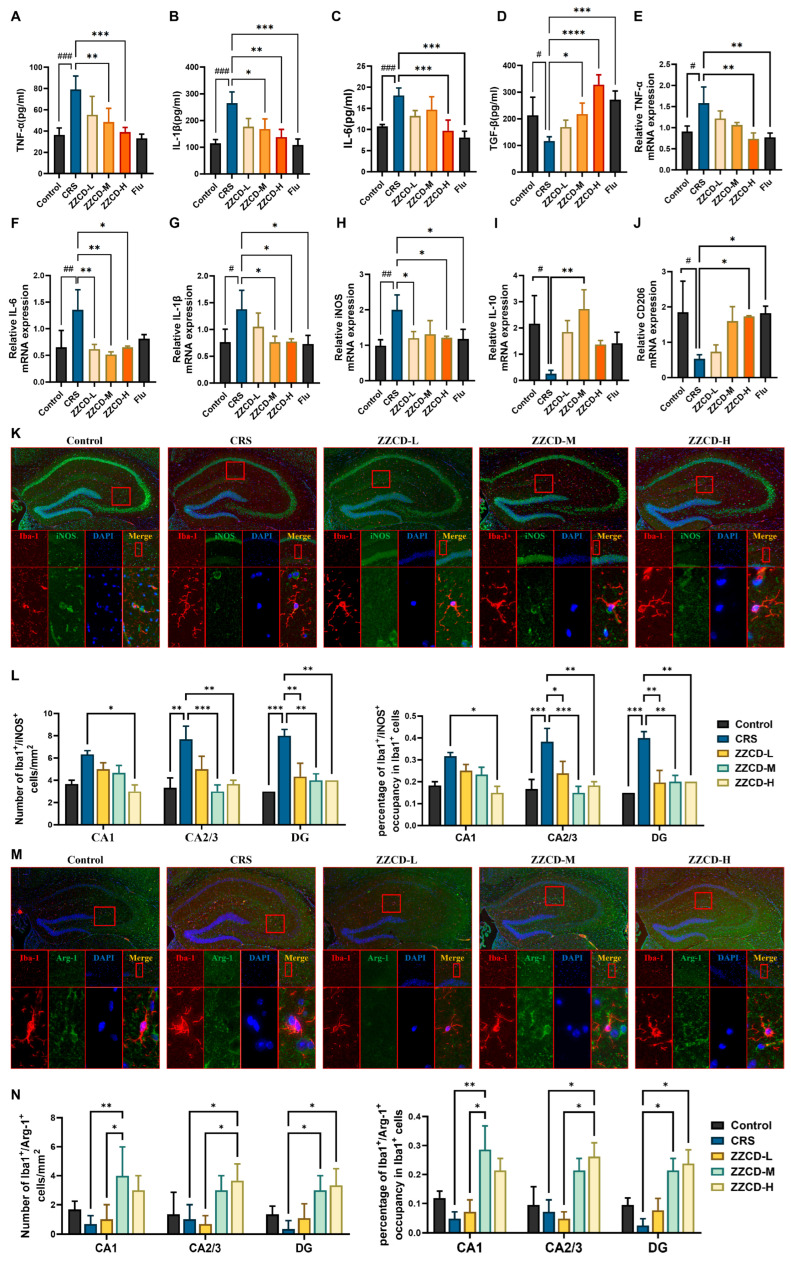
ZZCD alleviates neuroinflammation and mediates the transformation of M1 to M2 microglia. (**A**–**D**) The levels of TNF-α, IL-1β, TGF-β, and IL-6 in the serum of Con, CRS, ZZCD-L, ZZCD-M, ZZCD-H, and Flu group mice. (**E**–**J**) Gene expression levels of TNF-α, IL-6, IL-1β, TGF-β, iNOS, IL-10, and CD206 in brain tissue from Con, CRS, ZZCD-L, ZZCD-M, ZZCD-H, and Flx group mice. (**K**) Representative photomicrographs of M1 microglia immunofluorescence staining in the hippocampus. Representative confocal images of Iba1 (red), iNOS (Inducible nitric oxide synthase, green), and DAPI (blue) staining in the hippocampus. cale bar = 200 μm. (**L**) Quantitative analyses of the numbers and proportion of Iba1^+^/iNOS^+^ cells among Iba1^+^ cells in the CA1, CA2/3, and DG regions. (**M**) Representative photomicrographs of M2 microglia immunofluorescence staining in the hippocampus. Representative confocal images of Iba1 (red), Arg-1 (green), and DAPI (blue) staining in the hippocampus. cale bar = 200 μm. (**N**) The numbers and the proportion of Iba1^+^/Arg-1^+^ cells among Iba1^+^ cells in the CA1, CA2/3, and DG regions. Data are expressed as mean ± standard error of mean (SEM, *n* = 4), and One-Way ANOVA was used to compare differences. ^#^ *p* < 0.05,^##^ *p* < 0.01, ^###^ *p* < 0.001, compared to the Control group; * *p* < 0.05, ** *p* < 0.01, *** *p* < 0.001, **** *p* < 0.0001, compared to the CRS group.

An enzyme-linked immunosorbent assay (ELISA) was used to quantify peripheral blood levels of TNF-α, IL-1β, IL-6, and TGF-β in mice to investigate whether ZZCD treatment modulates neuroinflammation in chronic restraint stress (CRS)-exposed mice. Chronic restraint stress significantly reduced the concentration of the anti-inflammatory cytokine TGF-β in peripheral blood, while concurrently increasing the levels of three pro-inflammatory cytokines (TNF-α, IL-1β, and IL-6). Following treatment with mid-to-high doses of ZZCD, the levels of the pro-inflammatory cytokines (TNF-α, IL-1β, and IL-6) decreased, whereas the level of the anti-inflammatory cytokine TGF-β increased (Figure 3A–D). These results suggest that ZZCD can regulate neuroimmune responses.

Subsequently, to further explore ZZCD’s effects on microglial polarization, dual immunofluorescence staining and qRT-PCR were performed to measure the expression of microglial M1 pro-inflammatory markers (TNF-α, IL-6, IL-1β, iNOS) and M2 anti-inflammatory markers (TGF-β, IL-10, CD206) in hippocampal tissue from each experimental group. The results showed that ZZCD reversed the CRS-induced changes in marker expression. It attenuated the CRS-mediated upregulation of TNF-α, IL-6, IL-1β, and iNOS, and restored the CRS-induced downregulation of TGF-β, IL-10, and CD206 (Figure 3E–J).

**Figure 4:**
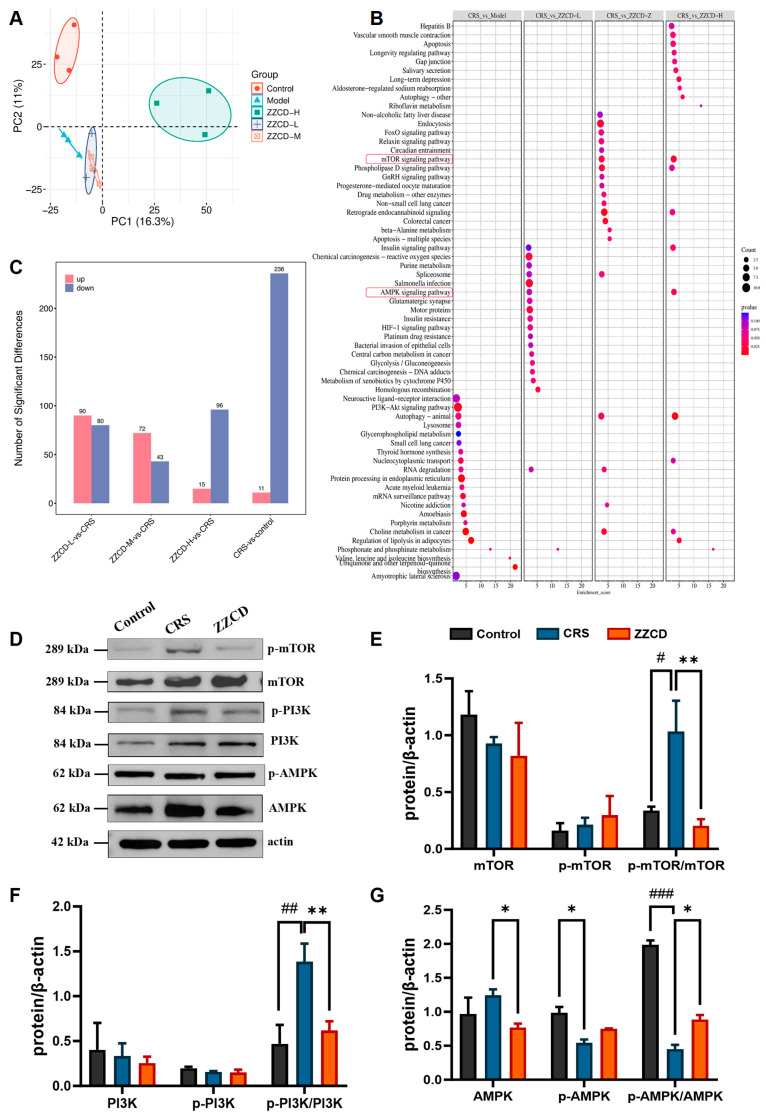
Proteomic changes in the brain tissue of CRS mice after ZZCD intervention. (**A**) Principal Component Analysis (PCA) of ZZCD regulatory proteins. (**B**) Enrichment analysis of KEGG pathways of different proteins between the ZZCD group and the CRS group. (**C**) The vertical bar of up-and down-regulated differentially expressed proteins (*n* = 3). (**D**–**G**) The expression of p-mTOR, mTOR, p-PI3K, PI3K, p-AMPK, and AMPK (*n* = 3). In comparison to the Control group, statistical significance was observed with ^#^ *p* < 0.05, ^##^ *p* < 0.01, and ^###^ *p* < 0.001; In contrast to the model group, statistical significance was observed with, ** *p* < 0.01, and * *p* < 0.05.

Additionally, CRS induced excessive microglial activation in the hippocampus (HPC). This activation was characterized by an amoeboid morphology, increased cell size, reduced branching processes, and a significant increase in the fluorescence intensity of ionized calcium-binding adapter molecule 1 (Iba1)—a marker of microglial activation (Figure 4A). Statistical analysis of co-immunofluorescence-stained cells revealed that the number of Iba1^+^/iNOS^+^ (M1-polarized) microglia in the CA1, CA2/3, and dentate gyrus (DG) subregions of the hippocampus was significantly higher in the CRS group than in the naïve control group (Figure 3K,L). In contrast, compared with the CRS group, the ZZCD-treated group exhibited a significant reduction in the number of Iba1^+^/iNOS^+^ microglia in the CA2/3 and DG subregions. Concurrently, the proportion of Iba1^+^/arginase-1^+^ (Arg-1^+^; M2-polarized) microglia among total Iba1^+^ microglia was significantly increased in the CA1, CA2/3, and DG subregions of ZZCD-treated mice (Figure 3M,N). Collectively, these findings demonstrate that ZZCD exerts anti-neuroinflammatory effects by increasing the proportion of M2-type anti-inflammatory microglia in the hippocampus.

### 2.4. ZZCD Alleviates Anxiety-like Behavior in Mice Through the mTOR Signaling Pathway

Subsequently, proteomic analysis was performed on brain tissue samples from the control and CRS model groups, resulting in the identification of approximately 5854 proteins. Principal component analysis (PCA) clustering successfully distinguished between brain tissue samples from naïve control mice, CRS-exposed model mice, and CRS-exposed mice treated with different doses of ZZCD (Figure 4A). To identify differentially expressed proteins (DEPs), the proteomic data were normalized using a linear function. After linear adjustment of the quantitative datasets, a total of 643 DEPs were identified. Compared with the CRS model group, the naïve control group had 236 downregulated proteins and 11 upregulated proteins. In contrast, compared with the CRS model group, the high-dose, low-dose, and mid-dose ZZCD groups exhibited 90, 72, and 15 upregulated proteins, respectively, and 80, 43, and 96 downregulated proteins, respectively (Figure 4B).

Functional enrichment analysis was further performed on these differentially expressed proteins (Figure 4C). KEGG pathway enrichment analysis showed that the DEPs in the CRS-vs-Control group, CRS-vs-ZZCD-M group, and CRS-vs-ZZCD-H group were enriched in the PI3K-Akt signaling pathway. The DEPs in the CRS-vs-ZZCD-M group and CRS-vs-ZZCD-H group were enriched in pathways including the mammalian target of rapamycin (mTOR) signaling pathway and the adenosine 5′-monophosphate-activated protein kinase (AMPK) signaling pathway.

Western blot analysis was conducted to validate the proteomic findings. Compared with the naïve control group, the CRS model group showed a significant increase in the phosphorylation level of phosphatidylinositol 3-kinase (PI3K; represented as p-PI3K/total PI3K ratio). Treatment with ZZCD significantly inhibited PI3K activation. Since mTOR is a key downstream effector protein of PI3K [28], we further analyzed mTOR phosphorylation. Compared with the CRS model group, the ZZCD-treated group significantly inhibited mTOR activation (as reflected by reduced p-mTOR protein levels). It decreased the p-AKT/AKT ratio (Figure 4E–G). Additionally, the CRS model group exhibited reduced phosphorylation of AMPK (p-AMPK), whereas the high-dose ZZCD group showed increased p-AMPK levels (Figure 4G). Collectively, the Western blot results showed that CRS significantly inhibited AMPK phosphorylation, and ZZCD reversed this inhibition. These findings suggest that ZZCD exerts regulatory effects on both the PI3K/mTOR and AMPK signaling pathways in CRS-exposed mice.

### 2.5. ZZCD Reverses Prebiotic Metabolites in the Intestine and Serum of CRS Mice to Alleviate Depression-like Behavior

**Figure 5:**
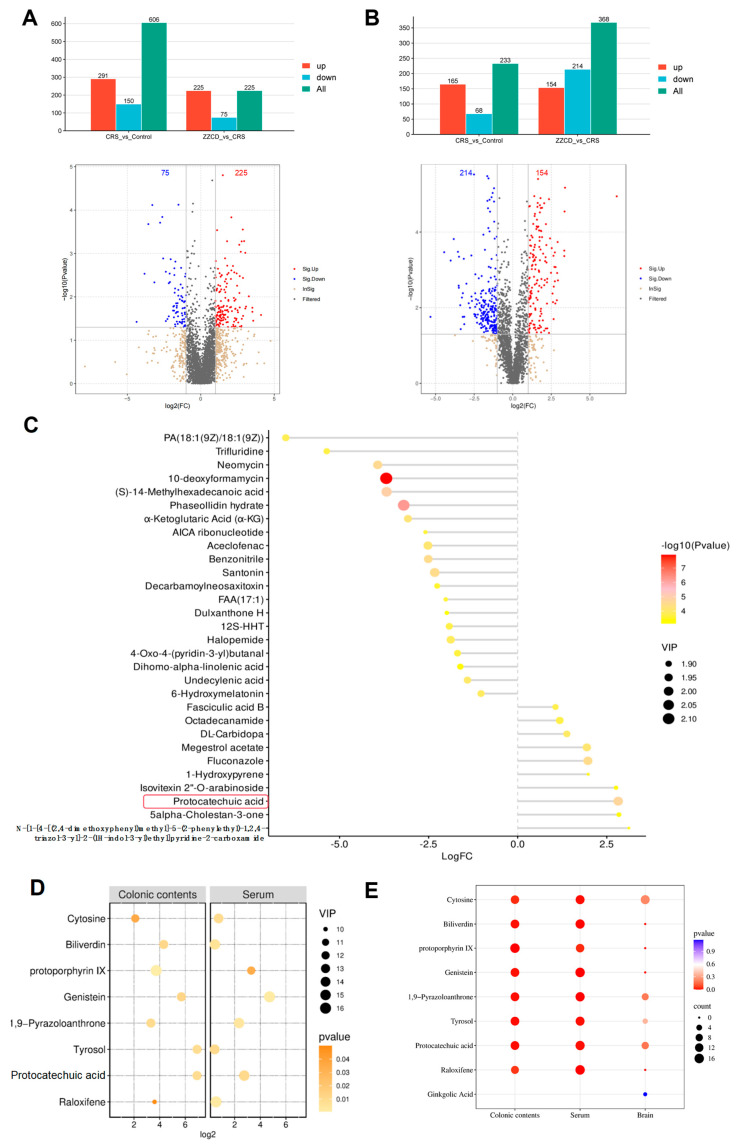
ZZCD affects metabolites associated with gut microbiota in CRS mice. (**A**) The number of differential metabolites in the colon contents between the CRS group and ZZCD-H and Control group. (**B**) The number of differential metabolites in the serum between the CRS group and ZZCD-H and the Control group. (**C**) The top 30 differential metabolites between the CRS group and the ZZCD-H group. (**D**) Quantification of protocatechuic acid (PCA) levels in serum and colonic contents. (**E**) Quantification of protocatechuic acid (PCA) levels in serum, colonic contents, and brain. *n* = 6, data are expressed as mean ± S.E.M.

Metabolomic analysis of colonic contents revealed that 897 metabolites in chronic restraint stress (CRS)-exposed mice exhibited significant differences compared to naïve control mice, in which 291 were upregulated, and 606 were downregulated (Figure 5A). Treatment with ZZCD significantly reversed the levels of 225 of these differentially expressed metabolites, including 150 upregulated and 75 downregulated metabolites (Figure 5B), of which neomycin, arabinose, 10-deoxyformycin, melibiose, hydron, threonic acid, 7-methylxanthine, glyoxylic acid, and protocatechuic acid in the top 30 differentially regulated metabolites were significantly upregulated in the ZZCD-treatment group (Figure 5C).

Similarly, 897 serum metabolites in chronic restraint stress (CRS)-exposed mice showed significant differences compared to naïve control mice, with 291 metabolites upregulated and 606 downregulated (Figure 5B). ZZCD treatment altered the levels of 368 metabolites, including 154 upregulated and 214 downregulated metabolites. Additionally, the highly upregulated metabolites in colonic contents, including cytosine, biliverdin, protoporphyrin IX, genistein, 1,9-pyrazoloanthrone, tyrosol, protocatechuic acid, and raloxifene, were also significantly enriched in the ZZCD treatment serum metabolome (Figure 5D). To verify whether these enriched metabolites can penetrate the blood–brain barrier, their levels were determined in colonic contents, serum, and brain tissue. The results showed that cytosine, 1,9-pyrazoloanthrone, and protocatechuic acid were significantly enriched in the brain (Figure 5E and Figure S1, and Tables S2–S4). These results indicate that ZZCD could increase some prebiotic metabolites to ameliorate anxiety- and depression-like behaviors in CRS-exposed mice.

**Figure 6:**
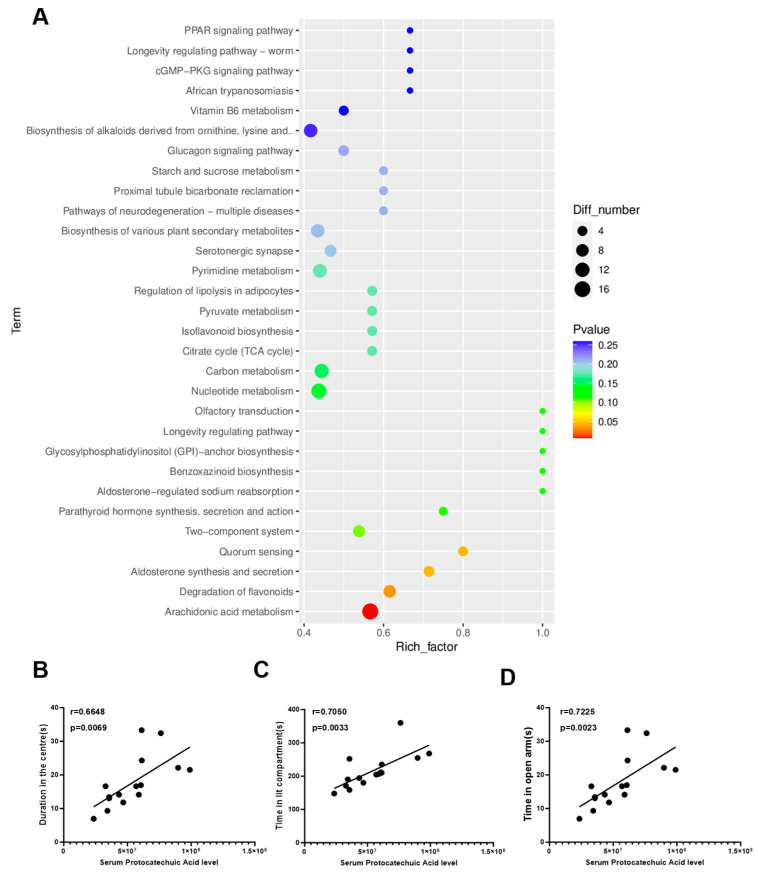
KEGG pathway enrichment analysis of colonic differential metabolites in CRS mice following ZZCD intervention. (**A**) KEGG pathway enrichment analysis of differential metabolites in colonic contents. (**B**–**D**) Correlation analysis between serum and colonic contents protocatechuic acid levels and behavioral indicators, such as time spent in the central area in the OFT, time spent in the light box in the LDB test, and time taken to enter the open arm in the EPM test. brain. *n* = 6, data are expressed as mean ± S.E.M.

KEGG pathway enrichment analysis of colonic differential metabolites revealed that ZZCD significantly modulated multiple interconnected biological pathways in CRS mice (Figure 6A). Arachidonic acid metabolism emerged as the most significantly enriched pathway, indicating attenuated pro-inflammatory eicosanoid synthesis, alongside flavonoid degradation, which supports the microbial biotransformation of herbal constituents into bioactive phenolics such as PCA. Quorum sensing and energy metabolism-related pathways, including the TCA cycle, pyruvate metabolism, and carbon metabolism, were also notably enriched, reflecting restored microbiota–host crosstalk and mitochondrial energy homeostasis. Furthermore, correlation analysis demonstrated that serum PCA levels positively correlated with the duration in the central zone of the open field test, time in the lit compartment of the light–dark box, and time in the open arms of the elevated plus maze, collectively establishing PCA as a critical gut-derived metabolite that bridges intestinal metabolic reprogramming with the alleviation of depression- and anxiety-like phenotypes (Figure 6B–D).

### 2.6. ZZCD Reverses Probiotics in the Intestine of CRS Mice to Alleviate Depression-like Behavior

**Figure 7:**
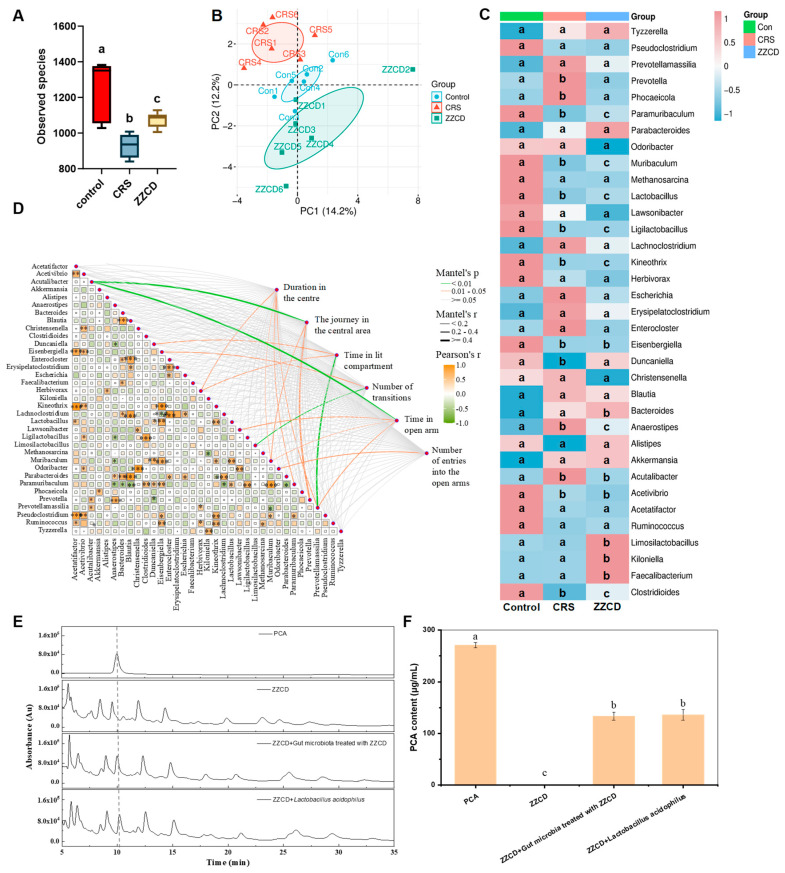
ZZCD ameliorated gut microbiota dysbiosis of CRS mice. (**A**) Alpha diversity index of the Control vs CRS group and CRS vs ZZCD group. (**B**) Principal coordinates analysis (PCoA) plots for the Control vs. CRS group and CRS vs. ZZCD group. (**C**) Top 30 species heat map showing inter-species differences at the genus level between CRS and ZZCD groups. The letters denote the results of inter-group significance testing, with a *p*-value threshold of <0.05. Tukey’s HSD test was employed to assess inter-group differences; different letters above each sample group indicate statistically significant differences between groups. (**D**) Mantel’s analysis was performed to examine the relationships between differential bacterial genus and behavioral indices, including the time and distance spent in the central area in the OFT, the time and number of entries spent in the light box in the LDB test, and the number of entries and time taken to enter the open arms in the EPM test. *** *p* < 0.001, ** *p* < 0.01, and * *p* < 0.05. (**E**,**F**) The PCA content detection in ZZCD treated with gut microbiota and *Lactobacillus acidophilus*.

To investigate the effect of ZZCD on gut microbiota, 16S rRNA gene sequencing was performed on fecal samples from the naïve control group, chronic restraint stress (CRS) model group, and ZZCD-treated groups, followed by differential microbiota analysis. Compared with the naïve control group, the CRS group exhibited a significant reduction in the Alpha diversity index, indicating decreased gut microbial biodiversity and richness in CRS-exposed mice. In contrast, the high-dose ZZCD group (ZZCD-H) reversed this reduction (Figure 7A). Principal coordinate analysis (PCoA) clearly distinguished gut microbiota profiles between the control and CRS groups, as well as between the ZZCD and CRS groups, highlighting the significant shifts in gut microbiota induced by CRS and the corrective effect of ZZCD treatment (Figure 7B).

At the genus level, compared to the native control, the CRS model group showed higher abundances of *Prevotella* and *Acutalibacte*, but lower abundances of *Paramuribaculum*, *Muribaculum*, *Lactobacillus*, *Kineothrix*, *Eisenbergiella*, *Duncaniella*, *Clostridioides*, *Anaerostipes*, and *Acetivibrio* (Figure 6C). ZZCD can reverse the effect of CRS on gut microbiota composition, significantly recovering the abundances of *Paramuribaculum*, *Muribaculum*, *Lactobacillus*, *Kineothrix*, *Eisenbergiella*, *Duncaniella*, *Clostridioides,* and *Anaerostipes* (Figure 7D). Furthermore, Mantel’s correlation analysis showed that the relative abundance of *Paramuribaculum, Muribaculum*, *Lactobacillus*, *Kineothrix*, and *Duncaniella* was significantly correlated with behavioral data, including distance traveled in the central area, time spent in the light box, and latency to enter the open arms (Figure 7D). Additionally, the transformation assay found that gut microbiota and *Lactobacillus acidophilus* can transform ZZCD to PCA (Figure 7E,F). These results demonstrate that ZZCD may modulate gut microbiota composition and microbe-derived metabolites in CRS-exposed mice, thereby alleviating anxiety- and depression-like behavior by regulating specific bacterial genera.

### 2.7. ZZCD-Derived Protocatechuic Acid from Gut Microbe Alleviates Anxiety-like Behavior in CRS Mice

**Figure 8:**
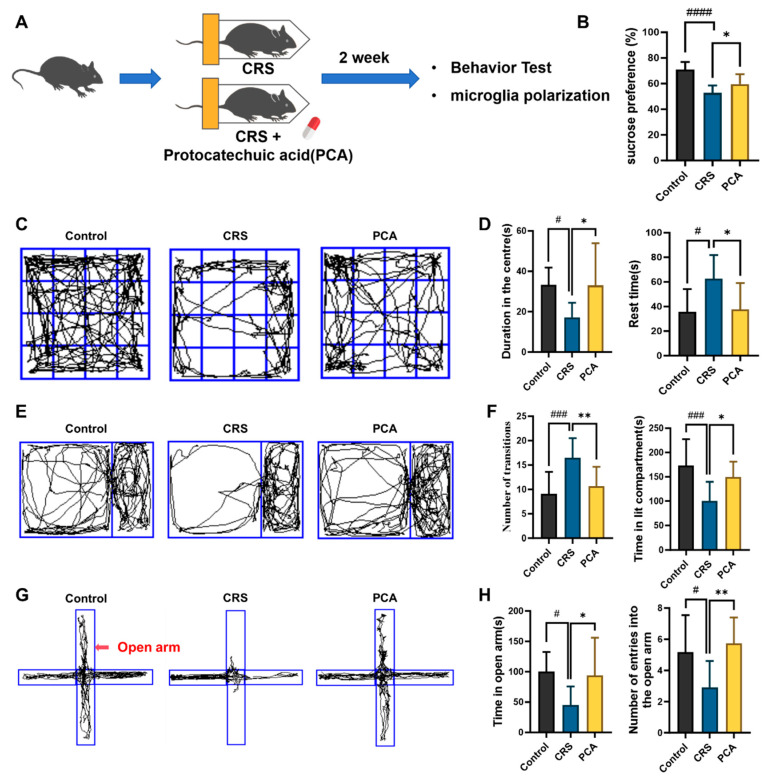
Supplementation with protocatechuic acid can correct depression- or anxiety-like behavior in CRS mice. (**A**) Schematic of the experimental paradigm. (**B**) Mean sucrose preference (%) in the sucrose preference test (SPT). (**C**) Movement trajectory of the mice in the Con, CRS, and Protocatechuic Acid (PCA,10 mg/kg) groups in OFT. (**D**) Rest time and the time spent in the central zone of the open field test (OFT). (**E**) Comparison of movement trajectory in LDB. (**F**) The amount of time mice spent in the light box and the number of times they switched between the light and dark boxes. (**G**) Comparison of movement trajectory in EPM with the vertical grid on the open arm. (**H**) The amount of time mice spent in the open arm and the number of times they entered the open arm. The lines represent the movement trajectories of the mice.Data are expressed as mean ± SEM. *n* = 12 mice per group. ^#^ *p* < 0.05, ^###^ *p* < 0.001, ^####^ *p* < 0.0001, compared to the Control group; * *p* < 0.05, ** *p* < 0.01, compared to the CRS group.

We conducted in vivo experiments to examine the potential therapeutic effects of protocatechuic acid, produced by gut microbes from ZZCD, on depressive-like behavior in CRS mice. Throughout the CRS model and subsequent behavioral testing, mice subjected to CRS received daily oral administration of protocatechuic acid (Figure 8A). After 14 days of PCA supplementation, CRS mice underwent a series of behavioral tests, including SPT, OFT, EPM, and LDB.

In the sucrose-preference test, PCA administration elicited a marked restoration of sucrose preference relative to vehicle-treated CRS-exposed mice, signifying a pronounced alleviation of anhedonic behaviors (Figure 8B). In the OFT, PCA-supplemented mice spent more time in the central area and less time resting (Figure 8C,D), reflecting improved exploratory behavior. In the LDB, the PCA treatment group showed reduced shuttling between the two boxes, accompanied by increased activity time in the light box (Figure 8E,F). In the EPM, the PCA treatment group showed increased visit frequency and duration to the open arms (Figure 8G,H), indicating reduced fear behavior. These behavioral improvements suggest that PCA may have anxiolytic and antidepressant effects on CRS mice.

### 2.8. ZZCD-Derived Protocatechuic Acid from Gut Microbe Regulates Microglial Cell Phenotype in CRS Mice Through the PI3K/Akt/mTOR Axis

**Figure 9:**
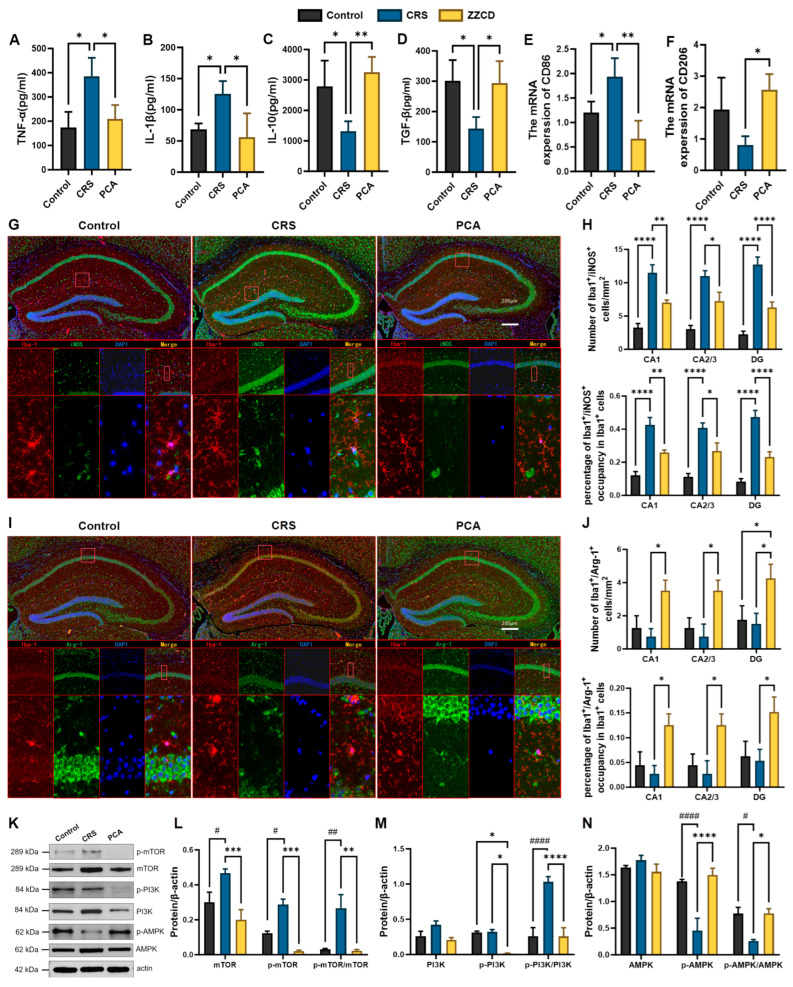
Supplementation with protocatechuic acid restores M1 polarization of hippocampal microglia in CRS mice. (**A**–**D**) The levels of TNF-α, IL-1β, IL-10, and TGF-β in the serum of Con, CRS, and PCA (10 mg/kg) group mice. (**E**,**F**) Gene expression levels of *CD86* and *CD206* in brain tissue from Con, CRS, and PCA (10 mg/kg) group mice. (**G**) Representative photomicrographs of M1 microglia immunofluorescence staining in the hippocampus. (**H**) Representative photomicrographs of M2 microglia immunofluorescence staining in the hippocampus. (**I**) The number of Iba1^+^/iNOS^+^ cells or Iba1^+^/Arg-1^+^ cells among Iba1^+^ cells in the CA1, CA2/3, and DG regions. Representative confocal images of Iba1 (red), iNOS (green), Arg-1 (green), and DAPI (blue) staining in the hippocampus. Scale bar = 200 μm. (**J**–**N**) The expression of p-mTOR, mTOR, p-PI3K, PI3K, p-AMPK, and AMPK (*n* = 3). Data are expressed as mean  ±  standard error of mean (SEM) (*n*  =  3 per group), and One-Way ANOVA compared differences. ^#^ *p* < 0.05, ^##^ *p* < 0.01, ^####^ *p* < 0.0001, compared to the Control group; * *p* < 0.05, ** *p* < 0.01, *** *p* < 0.001, **** *p* < 0.0001, compared to the CRS group.

Furthermore, compared with the CRS model group, the protocatechuic acid (PCA)-treated group exhibited significantly reduced levels of M1 pro-inflammatory phenotype markers, including TNF-α, IL-1β, and CD86. In contrast, the levels of M2 anti-inflammatory phenotype markers, including TGF-β, IL-10, and CD206, were significantly increased (Figure 9A–F). Immunofluorescence staining results further confirmed this phenotypic shift. Compared with the untreated CRS group, PCA treatment significantly reduced the number of Iba1^+^/inducible nitric oxide synthase^+^ (iNOS^+^) microglia (M1-polarized) and increased the number of Iba1^+^/arginase-1^+^ (Arg-1^+^) microglia (M2-polarized) in the hippocampus (Figure 9G–L). Collectively, these findings demonstrate that PCA not only ameliorates CRS-induced anxiety- and depression-like behaviors but also significantly attenuates the pro-inflammatory phenotype of hippocampal microglia.

Compared with CRS-mice, PCA significantly reduced the expression of phosphorylated (p)-PI3K and p-mammalian target of rapamycin (p-mTOR) in hippocampal microglia (Figure 9J–L). Additionally, PCA also significantly increased the expression of adenosine 5′-monophosphate-activated protein kinase (AMPK) and its phosphorylated form (p-AMPK) (Figure 9J,M). This finding suggests that PCA may regulate microglial activity and inflammation via the PI3K/mTOR/AMPK pathway, thereby mediating its effects on depressive-like symptoms.

## 3. Discussion

Zhi-Zi-Chi Decoction (ZZCD), a classic TCM formula from *Shanghan Lun*, is anchored in the TCM principle of “clearing heat and relieving restlessness” to target “heart-fire hyperactivity”, a syndrome linked to mental disturbances like irritability and depression. In TCM pathophysiology, “heat evil” accumulation in the “heart meridian” disrupts the “spirit” to “mental depression”, which is similar to modern mental illness (major depressive disorder, MDD). The formula’s “monarch-minister” composition, *Gardeniae Fructus* (Zhizi, monarch) for clearing heart/liver fire and resolving stasis, and *Semen Sojae Praeparatum* (Douchi, minister) for dispersing stagnated heat and protecting the spleen–stomach, reflects TCM’s holistic approach to restoring balance. This framework gains robust support from the present study’s results and recent literature. Chronic Restraint Stress (CRS) induced a “heat evil” state, as evidenced by elevated peripheral (serum TNF-α: 2.1-fold increase vs. control) and central (hippocampal IL-6: 2.3-fold increase) neuroinflammation, consistent with modern findings that neuroinflammation drives depression via microglial activation [29]. At the same time, ZZCD reversed these phenotypes (Figure 3). Mechanistically, ZZCD suppressed M1 microglial polarization and promoted M2 polarization (Figure 3), a biological interpretation of TCM’s “heat-clearing” that aligns with recent work showing M2 microglia secrete anti-inflammatory cytokines to restore synaptic plasticity [30].

Notably, ZZCD’s simultaneous modulation of gut microbiota, metabolites, and central signaling pathways mirrors TCM’s “holistic regulation” concept, a concept increasingly validated by literature on the gut–brain axis (GBX). Recent studies [31] have shown that TCM formulas often act via the GBX to normalize neuroinflammation, and our results extend this by demonstrating ZZCD’s ability to restore GBX balance: a key TCM “root cause” strategy that complements modern targeted therapies.

In recent years, the antidepressant potential of ZZCD has garnered increasing attention. Preclinical studies have demonstrated that ZZCD ameliorates depressive-like behaviors in chronic unpredictable mild stress (CUMS) models [32,33] through diverse mechanisms, including modulation of mitochondria-associated membrane integrity [17], restructuring of gut microbiota with subsequent enrichment of short-chain fatty acids (e.g., butyrate) [23], and regulation of the PI3K/AKT/mTOR signaling cascade [34]. Furthermore, geniposide, the principal bioactive constituent of Gardeniae Fructus, has been reported to exert neuroprotective effects by suppressing neuroinflammation and restoring neuroplasticity-related signaling in lipopolysaccharide-induced depressive models [22]. Nevertheless, these investigations have largely focused on isolated pathways or single herbal components, leaving a critical gap in understanding how gut microbiota-derived metabolites function as key effector molecules mediating the antidepressant efficacy of the complete formula. A systematic elucidation of the microbiota–metabolite–central signaling axis, therefore, remains urgently needed.

The present study used UHPLC-Q-TOF/MS to identify 424 constituents in ZZCD, with prenol lipids, organooxygen compounds, and flavonoids as the dominant classes, consistent with recent metabolomic studies on ZZCD [35]. A critical finding in our research is the identification of protocatechuic acid (PCA), a ZZCD-mediated metabolite, as a key “effector molecule.” Metabolomic analysis showed that ZZCD increased colonic PCA by 2.3-fold and serum PCA by 1.8-fold, extending recent findings that TCM formulas mediate intestinal metabolites to regulate disease [36]. Exogenous PCA recapitulated ZZCD’s antidepressant effects, including restoring sucrose preference and suppressing neuroinflammation (Figure 7 and Figure 8). Mechanistically, PCA inhibited PI3K/Akt/mTOR overactivation and activated AMPK, consistent with previous findings [37] which reported that PCA modulates these pathways to reduce neuroinflammation in Alzheimer’s disease models. This highlights that TCM formulas mediate alterations in intestinal metabolites as a critical step in ZZCD’s efficacy.

The gut microbiota–gut–brain (MGB) axis is a central mechanism of ZZCD’s neuroprotection, consistent with a surge of recent literature linking gut dysbiosis to neuropsychiatric disorders [38,39]. CRS disrupted gut homeostasis by reducing alpha diversity, increasing *Prevotella* and *Acutalibacter*, and decreasing beneficial *Lactobacillus* (Figure 6A–C). ZZCD reversed these changes by restoring the Shannon index to 91.5% of the control level, reducing *Prevotella* and enriching *Lactobacillus*. Mantel’s correlation analysis confirmed that *Lactobacillus* abundance correlated with improved behavior and reduced inflammation, supporting recent findings that *Lactobacillus* strains alleviate depression by producing SCFAs [40,41]. Additionally, untargeted metabolomics identified 897 differentially abundant metabolites in CRS mice, and ZZCD reversed these, including PCA, cytosine, and protoporphyrin IX (Figure 5C). These results indicate that ZZCD can restore intestinal microbiota to alleviate depression.

It is important to acknowledge that the neuroprotective roles of the gut microbiota–brain axis and protocatechuic acid (PCA) have been documented in prior literature. For instance, PCA has been shown to penetrate the blood–brain barrier after oral administration [42] and to exert anti-inflammatory and microglia-modulating effects within the central nervous system [43,44]. However, the present study makes incremental and distinct contributions through the following aspects: (1) it explicitly identifies PCA as a key microbiota-derived metabolite mediating the antidepressant effects of the classic TCM formula ZZCD, thereby shifting the focus from isolated compound activity to microbiota-mediated herbal formulation efficacy; critically, pharmacokinetic studies have demonstrated that PCA effectively penetrates the blood–brain barrier after oral administration [42], which substantiates its capacity to directly exert neuroprotective effects within the CNS; (2) it establishes an integrated mechanistic axis—the ZZCD–microbiota–PCA–neuroinflammation pathway—linking herbal medicine, gut microbial ecology, and central neuroimmune modulation into a coherent causal framework; and (3) it integrates behavioral, molecular biological, and metabolomic analyses within a unified herbal research framework, providing a multidimensional and systematic characterization of ZZCD’s therapeutic mechanism.

We identify the PI3K/Akt/mTOR and AMPK pathways as core axes of ZZCD’s neuroprotection, with PCA as a key mediator, aligning with recent advances in neuroinflammation signaling [45]. Pharmacokinetic studies have demonstrated that PCA effectively penetrates the blood–brain barrier, achieving therapeutically relevant concentrations in the central nervous system following oral administration [46]. CRS increased hippocampal p-PI3K and p-mTOR, driving M1 polarization and inhibiting neurogenesis, consistent with a previous study showing mTOR overactivation disrupts synaptic plasticity [47]. ZZCD and PCA reduced p-PI3K/PI3K by 42.3% and 39.1%, respectively, and p-mTOR/mTOR by 38.7% and 35.2%, restoring M2 polarization (Figure 4 and Figure 8). Notably, ZZCD exhibits bidirectional regulation, upregulating PI3K/Akt/mTOR in CUMS models [48] and downregulating it in CRS, mirroring TCM’s “balance restoration” and recent findings that TCM formulas normalize pathway activity rather than impose unidirectional effects [49]. Additionally, CRS reduced hippocampal p-AMPK, consistent with AMPK’s role as an energy sensor in neuroinflammation [50]. ZZCD and PCA increased p-AMPK by 56.8% and 51.3%, respectively (Figure 4 and Figure 8), consistent with previous work showing that AMPK activation alleviates depression by protecting mitochondria [51]. Proteomic analysis showed ZZCD-regulated proteins are enriched in both pathways (FDR 01), including PI3K, mTOR, and AMPK (Figure 4C).

## 4. Materials and Methods

### 4.1. Drugs

Gardeniae Fructus (Lot Number: 2103130) was obtained from Guiyang Jirentang (Guiyang, China). Semen Sojae Praeparatum (Lot Number: 2405011) was obtained from Hebei Baicao Kang Shen (Zhengzhou, China). Fluoxetine (Lot Number: F189157-5g), Protocatechuic Acid (Lot Number: P104383-250mg), and other inorganic reagents are all supplied by Shanghai Aladdin Bio-Chem (Shanghai, China).

### 4.2. Preparation and Characterization of ZZCD

The ZZCD, comprising 18 g of *Gardenia Fructus* (GF) and 8 g of *Semen Sojae Praeparatum* (SSP), was extracted according to traditional water decoction methods as following a standardized protocol [52]: First, 18 g of GF was decocted in 850 mL of deionized water under gentle reflux. This reflux process was maintained until the mixture volume was reduced to 500 mL. Subsequently, 8 g of SSP was added to the concentrated GF decoction, and the combined mixture was boiled continuously until the final volume reached 300 mL. The resulting ZZCD with a concentration of 0.6 g/mL was subsequently stored in a refrigerator at 4 °C for further use. Furthermore, we analyze the major components of the ZZCD using an ultrahigh-performance liquid chromatograph coupled with a quadrupole-time-of-flight tandem mass spectrometer (UHPLC-Q-TOF/MS) [53]. Specifically, a Vanquish Flex UHPLC chromatograph (Thermo Fisher Scientific, Inc., Waltham, MA, USA) equipped with an ACQUITY UPLC HSS T3 column (2.1 mm × 100 mm, 1.7 μm) (Waters, Corporation, Milford, MA, USA) was used for separation. The mobile phase consisted of water (0.1% formic acid) and acetonitrile, with a flow rate of 0.3 mL·min-1, a column temperature of 40 °C, and an injection volume of 6.0 µL. The MS data were collected using a hybrid quadrupole-orbitrap mass spectrometer (Q Exactive, Thermo Fisher Scientific, Inc., Waltham, MA, USA) equipped with a HESI-II spray probe. The parameters were set as follows: positive ion source voltage 3.7 kV, negative ion source voltage 3.5 kV, heated capillary temperature 320 °C, sheath gas pressure 30 psi, auxiliary gas pressure 10 psi, and desolvation temperature 300 °C.

The MS data were processed using Progenesis QI 3.0 (Waters Corp., MA, USA) with the following steps: raw data import, peak extraction, and adduct deconvolution. The identification was finally determined by considering the retention time error of the reference substance, mass error of mother ion, match degree of daughter ions, isotope distribution, and peak area after searching the reference substance database (TCM Pro 2.0, Beijing Hexin Technology Co., Ltd., Beijing, China.) and a theoretical database constructed by literature and public databases. The principal bioactive constituents of the ZZCD are shown in Figure 1 and Supplementary Table S1.

### 4.3. Animals

Healthy male C57BL/6 mice (8 weeks old, 20–25 g) were purchased from Speyford Laboratory Animal Co. Ltd. (Beijing, China; licence No. 110332251100006365) and habituated for seven days before study initiation in the SPF facility. Laboratory animals were housed under strictly controlled environmental conditions: a 12 h light/12 h dark cycle (lights on from 9:00 to 21:00), an ambient temperature of 23 ± 1 °C, and a relative humidity of 50% to 60%. Throughout the experimental period, animals had ad libitum access to filtered water and irradiated pellet feed. All experimental procedures involving animals were conducted in compliance with the Animal Care and Use Directive of the Chinese Academy of Chinese Medical Sciences (approval number: ERCCACMS21-2403-03; approval date: 26 March 2024).

Randomization and blinding: Mice were randomly allocated to experimental groups using computer-generated random numbers (Microsoft Excel 2019). The randomization sequence was generated by an independent researcher not involved in the experiments. Investigators performing behavioral tests, sample collection, and data analysis were blinded to group allocation throughout the study. Treatment codes were only revealed after statistical analysis was completed.

Sample size calculation: The sample size was determined based on our preliminary experiments using similar depression models [34]. A power analysis was conducted using G*Power 3.1 software (Heinrich Heine University Düsseldorf, Germany) [54] with the following parameters: effect size d = 0.8 (large effect according to Cohen’s conventions), α = 0.05, power (1 − β) = 0.8, and number of groups = 6. Based on this calculation, a minimum of 10 mice per group was required to detect significant differences in behavioral tests. We included 12 mice per group to account for potential attrition during the 14-day experiment.

Inclusion and exclusion criteria: All healthy male C57BL/6 mice aged 8 weeks with normal body weight (20–25 g) and behavior were included. Mice showing signs of illness, abnormal body weight (>20% deviation from group mean), or failure to adapt to the environment during the 7-day acclimatization period were excluded. During the experiment, mice exhibiting severe stress-induced self-injury, illness, or weight loss >25% would be excluded, though no mice met these criteria in this study.

Animal care and monitoring: Animals were monitored twice daily (morning and evening) for signs of distress, including weight loss, abnormal behavior, or physical injury. Humane endpoints were established as: (1) weight loss >25% of initial body weight, (2) severe self-injury or bleeding, (3) inability to eat or drink, or (4) signs of severe illness. No adverse events requiring intervention or euthanasia occurred during this study.

### 4.4. Chronic Restraint Stress Model and Treatment Regimens

The chronic restraint stress (CRS) mouse model was induced by confining each animal for 4 h a day in 50 mL breathable restraint tubes for 14 consecutive days. The Behavioral tests, physiological indicators, and molecular indicators were evaluated using the methods below.

A total of 72 mice were randomly divided into six experimental groups: vehicle-treated control group, CRS-exposed model group, fluoxetine (Flx)-treated positive control group (10 mg·kg^−1^·day^−1^), low-dose ZZCD-treated group (2 g·kg^−1^·day^−1^), mid-dose ZZCD-treated group (4 g·kg^−1^·day^−1^), and high-dose ZZCD-treated group (6 g·kg^−1^·day^−1^). The randomization was performed by an independent researcher not involved in the subsequent experiments. Cage location was randomized within the housing facility to minimize environmental confounders.

ZZCD extracts and PCA were administered by oral gavage (p.o.) at a volume of 10 mL/kg body weight (approximately 0.2 mL per mouse), once daily for 21 consecutive days. CRS exposure (4 h daily) was performed concurrently during the first 14 days of this treatment period. Body weight was monitored every 48 h throughout the experiment. After completion of the 14-day CRS exposure and the full 21-day treatment course, behavioral assays were conducted to assess depression-like and anxiety-like phenotypes. Immediately following the behavioral tests, mice were euthanized, and samples of serum, hippocampus, colon, and colonic contents were collected for subsequent histological examination, microbiome, proteome, and metabiotic analysis.

In the metabolite identification experiment, 18 mice were randomly divided into three groups (*n* = 6): naïve control, CRS model, and CRS+PCA (protocatechuic acid, 10 mg·kg^−1^·day^−1^). Following 14 days of intervention (consistent with the CRS exposure and treatment timeline described previously), behavioral tests were conducted to evaluate depression-like and anxiety-like phenotypes in the mice. Immediately after behavioral testing, all mice were euthanized. Serum samples and hippocampal tissue were then collected promptly for further experiments.

### 4.5. Animal Behavioral Tests

#### 4.5.1. Sucrose Preference Test (SPT)

The SPT was performed based on the previous study [55]. Post-CRS, mice were single-housed and habituated to two identical 50 mL bottles containing 1% sucrose for 24 h. During the subsequent 24 h test, one bottle contained 1% sucrose, and the other plain water; bottle positions were switched every 12 h to eliminate side bias. Sucrose preference was calculated as: [sucrose intake/(sucrose intake + water intake)] × 100%.

#### 4.5.2. Open Field Test (OFT)

General locomotion and anxiety were assessed 24 h after SPT in a square acrylic arena (50 cm base, 30 cm walls). Each mouse was released in the centre and its trajectory monitored for 6 min using Video analysis software (JLBehv-BWM-4, Keyexingcheng, Beijing, China).

#### 4.5.3. Light–Dark Box (LDB) Test

The apparatus consisted of two adjacent 25 cm^3^ Plexiglas chambers, one brightly illuminated and uncovered, the other dark and lidded, connected by a 3 cm aperture. Sixty min after the last treatment, mice were placed in the lit compartment [56]. entries and time spent in each zone were recorded for 5 min via JLBehv-BWM-4.

#### 4.5.4. Elevated Plus Maze (EPM) Test

The maze, elevated 70 cm above ground, comprised a 5 × 5 cm central platform flanked by two open (30 × 5 cm) and two enclosed (30 × 5 × 20 cm) arms. Animals were introduced onto the centre square and allowed to explore for 5 min; arm entries and duration were captured using JLBehv-BWM-4.

The investigators performing behavioral tests, sample collection, and data analysis were blinded to group allocation. An independent researcher assigned treatment codes and only revealed them after data analysis was completed.

### 4.6. ELISA

Enzyme-linked immunosorbent assay (ELISA) was performed according to the manufacturer’s instructions (Servicebio, Wuhan, China). The ELISA kits for TNF-α, IL-1β, IL-10, TGF-β, and IL-6 were used in the present work. Pre-coated 96-well plates (Servicebio) received 100 µL of serum sample/standard, or blank, incubated at 37 °C for 1 h, and washed (RT-3100C) 3 times. Biotinylated antibody (100 µL, 37 °C, 1 h) and streptavidin-HRP (100 µL, 37 °C, 30 min, dark) were added, each followed by three washes. TMB (100 µL, 10–30 min) was stopped with 2 M H_2_SO_4_; absorbance (450 nm) was read within 10 min (BioTek Instruments, Inc., Winooski, VT, USA.). Data were calculated from four-parameter logistic standard curves and expressed as pg mg^−1^ (tissue) or ng mL^−1^ (serum).

### 4.7. Real-Time Quantitative PCR (RT-qPCR) Detection

Total RNA was extracted from 5–20 mg hippocampal tissue using the KZ-5F-3D cryo-mill and RNA extraction reagent (G3013, Servicebio, Wuhan, China) with 3 mm zirconia beads. After complete homogenization, lysates were spun at 12,000× *g*, 4 °C, 10 min; 400 µL of the supernatant was mixed with chloroform substitute (G3014), centrifuged again, and RNA was precipitated with isopropanol at -20 °C for 15 min. The pellets underwent two washes with 75% ethanol, were air-dried, and subsequently dissolved in 15 µL of RNA solvent. Concentration and purity (A260/280) were assessed using a NanoDrop 2000 (Thermo Fisher Scientific, Inc., Waltham, MA, USA.); samples were standardized to 200 ng µL^−1^. cDNA was synthesized with 10 µL RNA, 4 µL 5× SweScript All-in-One RT SuperMix (G3337) Servicebio Technology Co., Ltd., Wuhan, China), and 1 µL gDNA remover in a 20 µL reaction: 25 °C 5 min, 42 °C 30 min, 85 °C 5 s. Each qPCR reaction (15 µL) comprised 7.5 µL of 2× Universal Blue SYBR Green Master Mix, 1.5 µL of 2.5 µM gene primers, 2 µL of cDNA, and 4 µL of nuclease-free water; three technical replicates were conducted on a Bio-Rad CFX Connect (Bio-Rad Laboratories, Inc., Hercules, CA, USA): 95 °C for 30 s, followed by 40 cycles of 95 °C for 15 s and 60 °C for 30 s, concluding with a melt curve from 65 to 95 °C in 0.5 °C increments. Relative expression was determined using the 2^(−ΔΔCt)^ technique.

### 4.8. Immunohistochemistry

Graded ethanol to water was used to rehydrate paraffin sections after they had been deparaffinized in an environmentally safe cleaning agent for three to ten minutes. Slides were cleaned with PBS (pH 7.4) (G0002, Servicebio, Wuhan, China) on a shaker for three to five minutes following heat-mediated antigen retrieval and chilling. The tissue was encircled with a hydrophobic pen and blocked with 3% BSA (GC305010, Servicebio, Wuhan, China) on a shaker (3 × 5 min) for 30 min. Sections were incubated overnight at 4 °C with a cocktail of two primary antibodies raised in distinct species (Servicebio, Wuhan, China). After 3 × 5 min PBS washes, species-matched fluorophore-conjugated secondary antibodies were applied and incubated at room temperature in the dark for 50 min. After further PBS rinses, nuclei were counterstained with DAPI (G1012, Servicebio, Wuhan, China). Endogenous autofluorescence was quenched with reagent B (G1401, Servicebio, Wuhan, China) on a shaker, and slides were rinsed under running water (10 min). Coverslips were mounted with antifade medium. Images were acquired using the following filter sets: DAPI (Ex 330–380 nm, Em 420 nm), 488 (Ex 465–495 nm, Em 515–555 nm), Cy3 (Ex 510–560 nm, Em 590 nm), and Cy5 (Ex 608–648 nm, Em 672–712 nm).

### 4.9. Proteome Analysis

Serum samples and hippocampal tissue from the chronic restraint stress model and treatment regimens were collected for proteome analysis; each treatment had three independent samples. Protein processing began with denaturation, reduction, alkylation, and proteolytic cleavage. Tissue powder was suspended in 200 µL UA buffer (8 M urea, 0.1 M Tris-HCl, pH 8.5) and homogenized by vortexing at 4000 rpm for 15 min at 4 °C. The lysate pH was adjusted to 7.0 with 100 mM NH_4_HCO_3_ before 0.05 M iodoacetamide was added; alkylation proceeded for 20 min in the dark. Trypsin (Promega, Corporation, Madison, WI, USA) was then introduced, and digestion was carried out for 2 h at 37 °C with gentle agitation (500 rpm), followed by an overnight step at 4 °C. The reaction was quenched with 1% formic acid (*v*/*v*). After centrifugation at 12,000× *g* for 5 min, the supernatant was loaded onto pre-conditioned SEP-PAK VAC C18 cartridges (Waters, Corporation, Milford, MA, USA.). Peptides were captured, washed with 0.1% formic acid, and eluted with 50% acetonitrile containing 0.1% TFA. The eluate was lyophilized and stored at −20 °C until analysis.

For DIA acquisition, dried peptides were re-dissolved in injection buffer and separated on an Acquity M-class UPLC (Waters) coupled to a ZenoTOF 7600 mass spectrometer (Sciex, Framingham, MA, USA) via an OptiFlow Turbo V source, controlled by SciexOS 3.1. Chromatographic and MS parameters were set according to the manufacturer’s recommended settings. Raw files were processed with DIA-NN (v1.8.1) for both identification and label-free quantification.

### 4.10. Western Blot Assay

Serum samples and hippocampal tissue from the chronic restraint stress model and treatment regimens were collected for Western blot analysis; each treatment had three independent samples. A BCA kit (Thermo Fisher Scientific) was used to measure the total protein in liver samples. Equivalent amounts were transferred to PVDF membranes at 240 mA for 90 min after being resolved on 12% SDS-PAGE gels. The membranes were blocked and then incubated overnight at 4 °C with the primary antibodies listed in Table S7. Following five washes with TBST (7 min each), blots were incubated for 1 h with HRP-conjugated goat anti-rabbit IgG (1:5000, MedChemExpress, Monmouth Junction, NJ, USA.). Immunoreactive bands were detected using ECL Prime reagent (Epizyme Biotech, Hangzhou, China) and analyzed densitometrically with ImageJ 1.52a (National Institutes of Health, Bethesda, MD, USA).

### 4.11. Microbial Diversity Analysis

The E.Z.N.A.^®^ Soil DNA Kit (Omega Bio-tek, Norcross, GA, USA) and the AxyPrep DNA Gel Extraction Kit (Axygen Biosciences, Union City, CA, USA) were employed to extract and purify microbial DNA from fresh mouse colonic material. The kits were used according to the manufacturer’s instructions. Using chemistry from the Sequencing Kit 2.0, we blunt-end-ligated amplicons into SMRTbell libraries (Pacific Biosciences of California, Inc., Menlo Park, CA, USA.) and sequenced them on PacBio Sequel II 8M cells. Shanghai Biozeron Biotechnology Co., Ltd. (Shanghai, China) conducted all amplicon sequencing. To obtain demultiplexed circular consensus sequence (CCS) data, we processed PacBio raw reads using SMRT Link Analysis version 9.0. The rarefaction study employed Mothur version 1.21.1 [57] to elucidate diversity indices, including Chao1, ACE, Simpson, and Shannon. The beta diversity analysis utilized the Euclidean distance matrix for principal component analysis (PCA) [58], and the Bray–Curtis or UniFrac distance matrices to evaluate the outcomes of principal coordinates analysis (PCoA) [59] and non-metric multidimensional scaling (NMDS) with the vegan community ecology package, R-forge (https://r-forge.r-project.org/) (accessed on 15 May 2025). Using the “RandomForest” package, we did Random Forest analyses to find the most important bacterial genera for each group [60]. The “ggplot2” and “pheatmap” packages were used to show the relative abundances of these key taxa and the accuracy of the Random Forest model, respectively. The Wilcoxon rank-sum (Mann–Whitney) test was employed to assess differences between two groupings, using the wilcox. test function in R, with a significance level of *p* ≤ 0.05.

### 4.12. Metabolomic Analysis

Frozen tissue (100 mg) was ground in liquid nitrogen, resuspended in 500 µL of ice-cold 80% methanol/0.1% formic acid, vortexed, incubated on ice for 5 min, and then centrifuged at 15,000× *g* for 5 min at 4 °C. The resultant supernatant was re-centrifuged (15,000× *g*, 4 °C, 10 min) and diluted with LC-MS-grade water to 53% methanol. A Vanquish UHPLC (Thermo Fisher Scientific, Dreieich, Germany) was coupled to an Orbitrap Q Exactive HF (Thermo Fisher, Germany) and utilized a Hypersil Gold column (100 × 2.1 mm, 1.9 µm) for the separation of metabolites at a flow rate of 0.2 mL min^−1^ and a temperature of 40 °C. The mobile phases consisted of 0.1% formic acid–water (A) or 5 mM NH_4_OAc pH 9 (A) and methanol (B), applied in a 17 min linear gradient from 2 to 100% B. MS operated in ±polarity (spray 3.2 kV, capillary 320 °C, sheath 40 arb, aux 10 arb). Raw files were processed using Compound Discoverer 3.1 (RT tolerance: 0.2 min; mass accuracy: 5 ppm; CD3.1, Thermo Fisher). Peaks were matched against mzCloud (https://www.mzcloud.org/) (accessed on 23 May 2025), mzVault, MassList, KEGG, HMDB, and LIPID Maps. Data were normalized to total ion intensity; differential metabolites were defined by VIP > 1, *p* < 0.05, and |FC| ≥ 2. PCA, PLS-DA, heatmaps, and pathway enrichment were performed in statistical software R (R version R-3.4.3), Python (Python 2.7.6 version), and CentOS (CentOS release 6.6). The content of metabolites was validated by UPLC methods by standards of cytosine, 1,9-pyrazoloanthrone, and protocatechuic acid(purchased from Shanghai Aladdin Bio-Chem Technology Co., Ltd., Shanghai, China).

### 4.13. Statistical Analysis

Outlier testing was performed using Grubbs’ test (or the interquartile range method, as appropriate for the dataset distribution) on all continuous data prior to statistical analysis. No significant outliers were detected, and therefore no data points were excluded from the reported datasets. All data were analyzed using GraphPad Prism software, 10.0 ( LLC, San Diego, CA, USA.) and expressed as mean ± standard deviation (SD). Inter-group comparisons were performed using one-way analysis of variance (ANOVA) followed by Bonferroni post hoc tests. Statistical significance was defined at *p* < 0.05.

## 5. Conclusions

Collectively, our data establish Zhi-Zi-Chi Decoction (ZZCD) as a multi-targeted, microbiota-directed therapy that alleviates chronic restraint stress (CRS)-induced anxiety and depression-like behaviors in mice. ZZCD drives the reprogramming of hippocampal microglia from a pro-inflammatory M1 to an anti-inflammatory M2 phenotype by increasing beneficial genera (i.e., *Lactobacillus*), reducing pro-inflammatory genera (i.e., Prevotella and Massilia), and enriching the prebiotic bioactive metabolite protocatechuic acid (PCA). Mechanistically, ZZCD and its gut microbiota-derived metabolites PCA exert bidirectional regulation on the PI3K/Akt/mTOR axis via suppressing excessive p-PI3K/p-mTOR activation and on the AMPK pathway via increasing p-AMPK levels. These findings identify PCA as a critical effector molecule mediating ZZCD’s therapeutic effects and underscore the clinical potential of targeting the gut–brain–immune–metabolic axis for the management of major depressive disorder (MDD).

While the present findings establish strong associations among gut microbiota remodeling, PCA enrichment, and behavioral improvements—supported by in vitro transformation experiments—direct causal links between microbiota alterations and neurobehavioral phenotypes remain to be definitively established. Future studies utilizing microbiota depletion, germ-free animals, or fecal microbiota transplantation are warranted to confirm that gut microbiota changes directly mediate the antidepressant effects of ZZCD.

## Data Availability

The original contributions presented in this study are included in the article/Supplementary Material. Further inquiries can be directed to the corresponding authors.

## Supplementary Materials

The following supporting information can be downloaded at: https://www.mdpi.com/article/10.3390/ph19060819/s1. Figure S1: The colon contents were analyzed using UPLC-QTF-MS/MS technology. (A) The total ion current (TIC) of the colon contents under positive ion scanning mode; (B) The total ion current (TIC) of the colon contents under negative ion scanning mode; Table S1: Analysis results of ZZCD components—Detailed table. Table S2: Analysis results of metabolic components of colon contents—Detailed table. Table S3: Analysis results of metabolic components of brain—Detailed table. Table S4: Analysis results of metabolic components of serum—Detailed table. Table S5. Primers and annealing temperature for RT-qPCR. Table S6. The antibody for Immunohistochemistry. Table S7: The antibody for western blotting.

## Abbreviations

ZZCD	Zhizi Chi Decoction
CRS	Chronic-restraint-stress
PCA	Protocatechuic acid
MDD	Major Depressive Disorder
IL-1β	Interleukin-1beta
IL-6	Interleukin-6
IL-10	Interleukin-10
IL-18	Interleukin-18
TNF-α	tumor necrosis factor alpha
TGF-β	Transforming growth factor (TGF)-beta
iNOS	inducible nitric oxide synthase
Arg-1	Arginase-1
HPC	Hippocampus
NLRP3	NLR Family Pyrin Domain Containing 3
BDNF	Brain-derived neurotrophic factor
MGB	Microbiota–gut–brain
UHPLC-Q-TOF/MS	Ultrahigh-performance liquid chromatography–quadrupole time-of-flight tandem mass spectrometry
SCFA	Short-chain fatty acid
SPT	Sucrose preference test
OFT	Open field test
LDB	Light–dark box test
EPM	Elevated plus maze test
ELISA	Enzyme-linked immunosorbent assay
Flx	Fluoxetine
AMPK	AMP-activated protein kinase
PI3K	Phosphatidylinositol 3-kinase
p-PI3K	Phosphorylation Phosphatidylinositol 3-kinase
mTOR	mammalian target of the rapamycin
p-mTOR	Phosphorylation of mammalian target of the rapamycin
ULK1	Unc-51-like autophagy activating kinase 1
