# Supplementary material for: Zhi-Zi-Chi Decoction Alleviates Depressive-like Behaviors by Regulating Gut Microbiota and Targeting the AMPK/PI3K-TOR Pathway via Its Metabolite Protocatechuic Acid
Source: Pharmaceuticals (Basel). 2026 May 23;19(6):819. doi: 10.3390/ph19060819 (PMC13304515; doi:10.3390/ph19060819)
Supplement: Supplementary file 1 [file pharmaceuticals-19-00819-s001.zip › Table S5-7.pdf]

**Table S5.** Primers and annealing temperature for RT-qPCR.

| Genes | Forward primer | Reverse primer | Annealing(°C) |
|---|---|---|---|
| **IL-6** | AGTTGTGCAATGGCAATTCTGA | CTCTGAAGGACTCTGGCTTTGTC | 60 |
| **IL-1β** | GTAATGAAAGACGGCACACCC | CAGGCTTGTGCTCTGCTTGTG | 60 |
| **TNF-α** | GTGCCTATGTCTCAGCCTCTTCTC | GTTTGTGAGTGTGAGGGTCTGG | 60 |
| **iNOS** | CAACAGGAACCTACCAGCTCACT | AGCCTGAAGTCATGTTTGCCG | 60 |
| **IL-10** | AATAAGCTCCAAGACCAAGGTGT | CATCATGTATGCTTCTATGCAGTTG | 60 |
| **CD86** | TTGGGCACAGAGAAACTTGATAG | TTCGGGTGACCTTGCTTAGAC | 60 |
| **CD206** | TTGGACGGATAGATGGAGGGT | CCATAGAAAGGAATCCACGCA | 60 |
| **GAPDH** | CCTCGTCCCGTAGACAAAATG | TGAGGTCAATGAAGGGGTCGT | 60 |

**Table S6.** The antibody for Immunohistochemistry.

| Primary antibodies | CAT | species | Dilution | Second antibody |
|---|---|---|---|---|
| **IBA1** | GB12105 | Mouse | 1:200 | Cy3 conjugated Goat Anti-mouseIgG(H+L) |
| **INOS** | GB11119 | Rabbit | 1:100 | Alexa Fluor 488 conjugated Goat Anti-Rabbit IgG (H+L) |
| **ARG1** | GB11285 | Rabbit | 1:100 | Alexa Fluor 488 conjugated Goat Anti-Rabbit IgG (H+L) |

**Table S7.** The antibody for western blotting.

| Antibody | CAT | Brand | Dilution |
|---|---|---|---|
| **Phospho-mTOR** | HY-P80469 | MedChemExpress | 1:1000 |
| **mTOR** | HY-P80231 | MedChemExpress | 1:1000 |
| **Phospho-PI3 Kinase** | HY-P80846 | MedChemExpress | 1:1000 |
| **PI3K** | HY-P80866 | MedChemExpress | 1:1000 |
| **Phospho-AMPK** | HY-P80791 | MedChemExpress | 1:1000 |
| **AMPK** | HY-P80541 | MedChemExpress | 1:1000 |
| **AKT1** | HY-p80535 | MedChemExpress | 1:1000 |
| **PDK1** | HY-p80272 | MedChemExpress | 1:5000 |
| **ACTIN** | Servicebio | GB15001 | 1:5000 |
