# Supplementary figures and images for: Zhi-Zi-Chi Decoction Alleviates Depressive-like Behaviors by Regulating Gut Microbiota and Targeting the AMPK/PI3K-TOR Pathway via Its Metabolite Protocatechuic Acid
Source: Pharmaceuticals (Basel). 2026 May 23;19(6):819. doi: 10.3390/ph19060819 (PMC13304515; doi:10.3390/ph19060819)

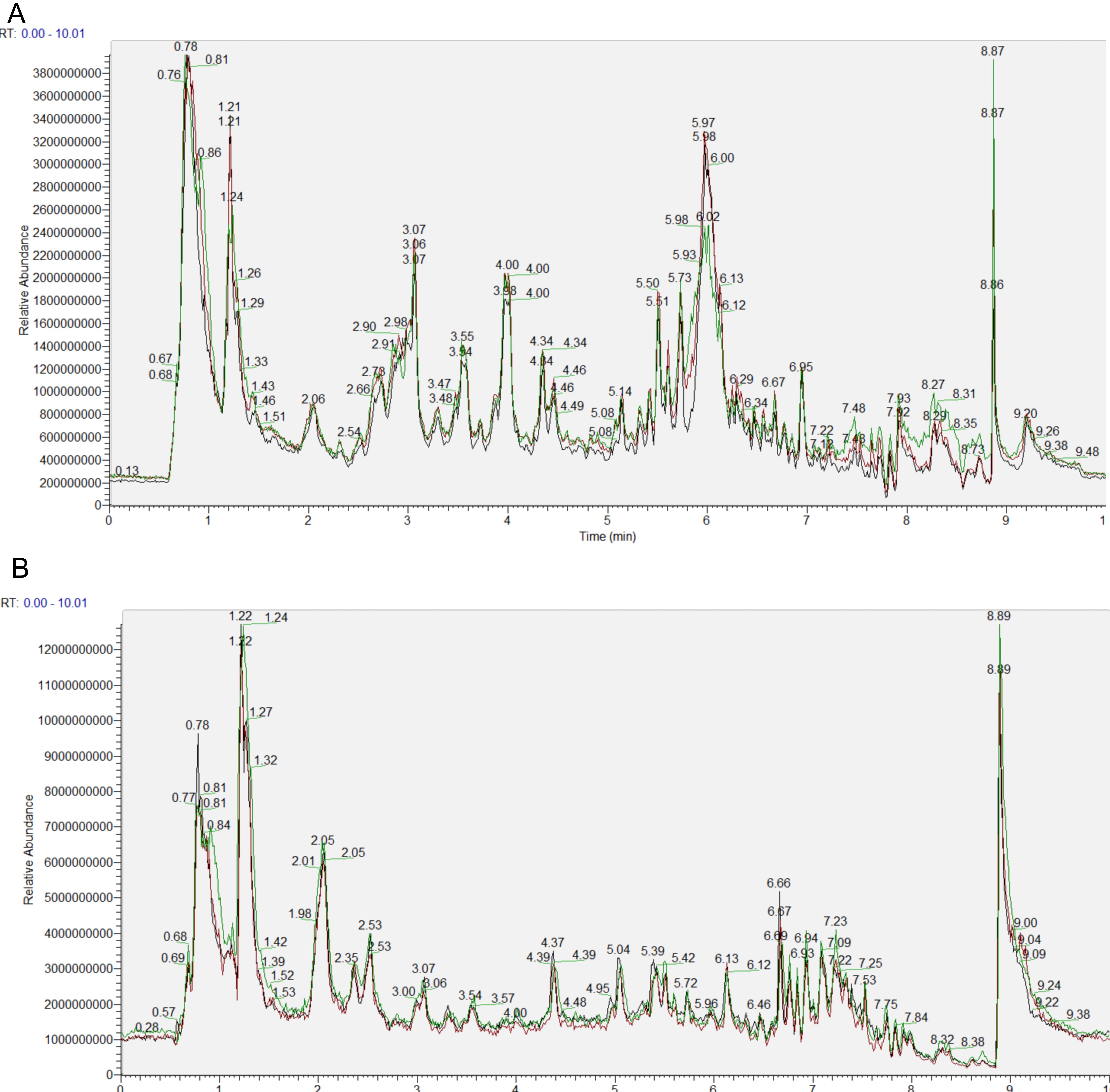

Supplement: Supplementary file 1 [file pharmaceuticals-19-00819-s001.zip › Supplementary Figure S1 .jpg]
